# Pathogenesis of local necrosis induced by *Naja atra* venom: Assessment of the neutralization ability of Taiwanese freeze-dried neurotoxic antivenom in animal models

**DOI:** 10.1371/journal.pntd.0008054

**Published:** 2020-02-07

**Authors:** Chien-Chun Liu, Yu-Shao Chou, Chun-Yu Chen, Kuei-Lin Liu, Guo-Jen Huang, Jau-Song Yu, Cho-Ju Wu, Geng-Wang Liaw, Cheng-Hsien Hsieh, Chun-Kuei Chen

**Affiliations:** 1 Molecular Medicine Research Center, Chang Gung University, Taoyuan, Taiwan; 2 Department of Emergency Medicine, En Chu Kong Hospital, New Taipei City, Taiwan; 3 Faculty of Biotechnology and Laboratory Science in Medicine, School of Medical Technology and Engineering, National Yang-Ming University, Taipei, Taiwan; 4 Department and Graduate Institute of Biomedical Sciences, College of Medicine, Chang Gung University, Taoyuan, Taiwan; 5 Department of Cell and Molecular Biology, College of Medicine, Chang Gung University, Taoyuan, Taiwan; 6 Liver Research Center, Chang Gung Memorial Hospital, Linkou, Taoyuan, Taiwan; 7 Research Center for Food and Cosmetic Safety, Research Center for Chinese Herbal Medicine, College of Human Ecology, Chang Gung University of Science and Technology, Taoyuan, Taiwan; 8 Department of Emergency Medicine, Chang Gung Memorial Hospital and Chang Gung University College of Medicine, Taoyuan, Taiwan; 9 Department of Emergency Medicine, Yeezen General Hospital, Taoyuan, Taiwan; 10 Graduate Institute of Clinical Medicine, College of Medicine, Taipei Medical University, Taipei, Taiwan; Texas A&M University Kingsville, UNITED STATES

## Abstract

*Naja atra* envenomation is one of the most significant clinical snakebite concerns in Taiwan. Taiwanese freeze-dried neurotoxic antivenom (FNAV) is currently used clinically for the treatment of cobra snakebite, and has been shown to limit the mortality of cobra envenomation to less than 1%. However, more than half of victims (60%) require surgery because of local tissue necrosis, a major problem in patients with cobra envenomation. Although the importance of evaluating the neutralizing effect of FNAV on this pathology is recognized, whether FNAV is able to prevent the local necrosis extension induced by *N*. *atra* venom has not been investigated in detail. Cytotoxins (CTXs) are considered as the major components of *N*. *atra* venom that cause necrosis. In the current study, we isolated CTXs from whole cobra venom and used both whole venom and purified CTXs to develop animal models for assessing the neutralization potential of FNAV against venom necrotizing activity. Local necrotic lesions were successfully produced in mice using CTXs in place of whole *N*. *atra* venom. FNAV was able to rescue mice from a subcutaneously injected lethal dose of cobra venom; however, it was unable to prevent CTX-induced dermo-necrosis. Furthermore, using the minimal necrosis dose (MND) of CTXs and venom proteome data, we found a dose of whole *N*. *atra* venom suitable for FNAV and developed a workable protocol for inducing local necrosis in rodent models that successfully imitated the clinical circumstance of cobra envenoming. This information provides a more comprehensive understanding of the pathophysiology of *N*. *atra* envenomation, and serves as a guide for improving current antivenom strategies and advancing clinical snakebite management in Taiwan.

## Author summary

*Naja atra* envenomation is an important public health issue in Taiwan. Although the mortality rate of cobra snakebite is controlled using antivenom, more than half of victims develop symptoms of local necrosis and require surgical intervention. Whether the Taiwanese freeze-dried neurotoxic antivenom (FNAV) currently in clinical use is able to prevent the local necrosis extension induced by *N*. *atra* venom is still unclear. In this study, we developed a dermo-necrosis animal model using purified cytotoxins (CTXs), the major necrosis-related proteins from *N*. *atra* venom. We found that FNAV was able to neutralize the lethality of whole cobra venom, but was unable to neutralize the necrosis induced by CTXs *in vivo*. This finding introduced an example that supplementary quality control assays may be necessary to determine the effectiveness of antivenoms in neutralizing specific pathology induced by the venom; only evaluating the rodent lethality prevention is insufficient. Our results provide insights that should help improve current antivenoms and advance cobra snakebite management in Taiwan.

## Introduction

Envenomation by snakebite is a significant global public health issue, but is a particularly important issue in tropical (subtropical) countries and some poor rural communities [1]. Epidemiological studies have estimated that at least 1,800,000 envenoming events and 82,000 associated deaths occur each year due to snakebite [2]. Because snakes are ectothermic animals, snakebite cases are particularly burdensome in regions with a warmer climate, such as South Asia, Southeast Asia, and Africa [2–4].

Taiwan (formerly known as Formosa) is a subtropical island located in East Asia that is home to more than 40 snake species [5], six of which are of highly venomous [6]. According to the World Health Organization (WHO) categorizations in the guideline for antivenom production, four of these venomous snakes—*Bungarus multicinctus*, *Naja atra*, *Trimeresurus stejnegeri*, and *Protobothrops mucrosquamatus*—belong to the category 1, whereas the other two—*Deinagkistrodon acutus* and *Daboia russelii siamensis*—belong to the category 2 [7]. There are approximately 1,000 envenoming incidents in Taiwan each year, about 23.5–36% of which are *N*. *atra* envenoming [6, 8].

The government of Taiwan has produced snake antivenom for more than nine decades. After a series of improvements and refinements in the production process, there are now four types of antivenoms against the six most clinically significant snakebites available for clinical use. All are in the form of lyophilized F(ab)_2_ from equine serum—two as bivalent antivenoms, and two as monovalent antivenoms [5, 9]. The first two are freeze-dried hemorrhagic antivenom (FHAV) against *T*. *stejnegeri* and *P*. *mucrosquamatus* and freeze-dried neurotoxic antivenom (FNAV) against *B*. *multicinctus* and *N*. *atra*, whereas the latter two are freeze-dried *D*. *acutus* antivenom against *D*. *acutus* and freeze-dried *D*. *russelii siamensis* antivenom against *D*. *russelii siamensis*. A complete antivenom system against all clinically significant snakebites would greatly aid in the control of snakebite-related mortality, which currently stands at less than 1% in Taiwan [8].

The cobra (genus *Naja*) is one of the most important venomous snakes that contribute to snakebite injury in Southeast Asia, including Taiwan [2, 10]. Cobra (*N*. *atra*) envenoming in Taiwan causes significant local tissue necrosis with swelling, in addition to inducing weak systemic neurotoxic effects [11, 12]. Although the bivalent FNAV, produced by the Centers for Disease Control, ROC (Taiwan), is clinically available for treatment of cobra envenoming, the surgical intervention rate still remains high (60–65%) [11, 13, 14]. Despite early antivenom administration (<6 h), more than half of cobra envenoming patients develop local tissue necrosis requiring debridement or other surgical intervention [11]; thus, limits to the ability of FNAV to neutralize the cytotoxicity of *N*. *atra* have been widely discussed.

Snake venom is a mixture of components. The major toxic proteins in *N*. *atra* venom are neurotoxins, phospholipase A_2_ (PLA_2_) proteins and cytotoxins (CTXs) [15], the latter of which have been reported to induce necrosis symptoms [16–18]. The potential of FNAV to neutralize *N*. *atra* venom is currently determined based on its ability to prevent lethality of *N*. *atra* venom in mice. However, whether FNAV is effective in neutralizing the local necrosis induced by *N*. *atra* venom has not been systematically investigated. In the present study, we purified and characterized the major components of *N*. *atra* venom and assessed the lethality and necrosis-promoting ability of whole venom and purified CTXs in animal models. The effectiveness of FNAV against the mortality and morbidity (necrosis) induced by *N*. *atra* venom was evaluated by pre-administration of venom proteins and antivenom.

## Materials and methods

### The snake venom and antivenom

The crude venom of *N*. *atra* was obtained from the world snake king education farm, Tainan, Taiwan. It was immediately lyophilized and stored at -20 °C until used. The FNAV (batch number: FN10201, FN10302 and FN10303) was purchased from Center of Disease and Control, R.O.C (Taiwan). The lyophilized antivenom powder was dissolved at 80 mg/ml in antivenom diluted buffer, providing with antivenom, for use in this investigation.

### C_18_ reverse-phase high-performance liquid chromatography (RP-HPLC) fractionation of *N*. *atra* venom

The venom of *N*. *atra* was separated by RP-HPLC, as previously described [19]. Briefly, crude venom (100 μg protein) was dissolved in solvent A (water containing 0.1% trifluoroacetic acid (TFA), and separated by RP-HPLC using a Supelco Discovery 300 Å C_18_ (4.6 × 150 mm, 3 μm particle size) column (Sigma-Aldrich, St. Louis, Missouri, USA). The flow rate was set to 0.7 mL/min, and the column was developed with a linear gradient of solvent A and solvent B (acetonitrile containing 0.1% TFA) as follows: isocratic 5% solution B for 3 minutes, followed by linear gradients of 5–10% solvent B for 2 minutes, 10–16% solvent B for 6 minutes, 16–28% solvent B for 4 minutes, 28–41% solvent B for 32 minutes, 41–52% solvent B for 5 minutes, 52–80% solvent B for 3 minutes, 80–100% solvent B for 1 minutes, 100% solvent B for 1 minutes, 100–5% solvent B for 2 minutes, and re-equilibration with 5% solvent B for 1 minutes. Peaks were detected by monitoring absorbance at 280 nm. Chromatographic fractions were collected manually. This process was performed to separate 15 mg of *N*. *atra* venom proteins. Then, fractions were lyophilized and stored at -20 °C for further analysis.

### Sodium dodecyl sulfate-polyacrylamide gel electrophoresis (SDS-PAGE)

The lyophilized fractions from RP-HPLC were dissolved by PBS. The protein concentration of each fraction was measured using the Pierce BCA Protein Assay Kit (Thermo Fisher Scientific, Waltham, Massachusetts, USA). Two microgram of each fraction proteins was analyzed by SDS-PAGE under reducing condition. Briefly, samples were dissolved in sample buffer (125 mM Tris, 25% glycerol, 10% 2-mercaptoethanol, 4% SDS, 0.05% bromophenol blue) and heated at 95 °C for 5 minutes. Samples were then loaded onto a 15% gel and further separated by SDS-PAGE. The location of proteins in SDS-PAGE gels was visualized by Coomassie Brilliant Blue staining.

### In-direct enzyme-linked immunosorbent assay (Indirect ELISA)

Each fraction of *N*. *atra* venom (50 ng) was diluted in 100 μl phosphate-buffered saline (PBS) and coated onto 96-well polystyrene microplates (Corning Inc., Corning, New York, USA) by incubating at 4°C overnight. The plates were washed six times with 200 μl of PBST (contain 0.1% Tween-20) and blocked with 200 μl of 1% ovalbumin in PBS at room temperature for 2 hours. After repeating the washing step for six times, FNAV (8 mg/ml) was diluted (1:5000) in PBS, and added to each well. After incubation at room temperature for 2 hours, the excess antibodies were removed by washing 6 times with PBST. Then, the alkaline phosphatase-conjugated secondary antibody (Santa Cruz Biotechnology, Dallas, Texas, USA) was diluted (1:5000) in PBS and added to each well and incubated at room temperature for 1 hour. After finally washing each well 6 times with PBST, the substrate 4-methyl umbelliferyl phosphate (100 μM, 100 μl/well) was added to each well and incubated for 10 minutes. The fluorescence was measured with a SpectraMax M5 microplate reader (Molecular Devices, Silicon Valley, CA, USA) at excitation and emission wavelengths of 355 and 460 nm, respectively.

### In-solution tryptic digestion of protein

For protein identification, the proteins in each fraction were subjected to tryptic digestion before liquid chromatography-tandem mass spectrometry (LC-MS/MS) analysis, as described previously [20]. Briefly, 1 μg of protein was reduced by incubation with 10 mM dithiothreitol (DTT) at 60 °C for one hour and alkylated by incubation with 30 mM iodoacetamide (IAM) at room temperature in the dark for 30 minutes. Samples were then equilibrated by incubation again with 10 mM DTT at room temperature for 10 minutes. Proteins were digested with freshly prepared trypsin solution containing 20 μg/mL of trypsin (Promega, Madison, WI, USA) in 50 mM ammonium bicarbonate at 37 °C for 16 hours, following heated sample at 100 °C for 10 minutes to denature the trypsin enzyme. A 96-well Oasis HLB μElution plate (Waters, Milford, Massachusetts, USA) was used to desalt each sample according to the manufacturer’s protocol. After the sample was desalted, the tryptic peptides were lyophilized by SpeedVac and stored at -20 °C before LC-MS/MS analysis.

### LC-MS/MS analysis

Each peptide sample was reconstituted with 0.1% formic acid (FA), and then analyzed on a nano-LC–LTQ-Orbitrap Hybrid Mass Spectrometer (Thermo Fisher, San Jose, CA, USA), as described previously [21]. Briefly, the sample was loaded across a trap column (Zorbax 300SB-C18, 0.3 × 5 mm; Agilent Technologies, Wilmington, DE, USA) at a flow rate of 0.2 μl/min in HPLC buffer (0.1% FA), and separated on a resolving 10-cm analytical C18 column (inner diameter, 75 μm) using a 15-μm tip (New Objective, Woburn, MA, USA). The peptides were eluted using a linear gradient of 0–10% HPLC buffer B (100% ACN containing 0.1% FA) for 3 minutes, 10–30% buffer B for 35 minutes, 30–35% buffer B for 4 minutes, 35–50% buffer B for 1 minute, 50–95% buffer B for 1 minute and 95% buffer B for 8 minute, with a flow rate of 0.25 μl/min across the analytical column. The resolution of the Orbitrap is 30,000, and the ion signal of (Si(CH_3_)_2_O)_6_H+ at 445.120025 (m/z) was used as a lock mass for internal calibration. A procedure that alternated between one MS scan followed by 10 MS/MS scans for the 10 most abundant precursor ions in the MS scan was applied. The m/z values selected for MS/MS were dynamically excluded for 180 seconds. For MS scans, the m/z value of the scan range was 400 to 2000 Da. For MS/MS scans, more than 1 × 10^4^ ions were accumulated in the ion trap to generate MS/MS spectra. Both MS and MS/MS spectra were acquired using one scan with maximum fill-times of 1000 and 100 ms for MS and MS/MS analysis, respectively. For database searching, MS raw data files were analyzed by Proteome Discoverer Software (version 1.4.1.14; Thermo Fisher, San Jose, CA, USA), and searched against other lobe-finned fish and tetrapod clade taxonomy in the Swiss-Prot database using MASCOT. The enzyme specificity parameter was set to “trypsin” and one missed cleavage was allowed. Carbamidomethylation of cysteine was set as a static modification and oxidation of methionine, acetyl (protein N-term) and Gln- > pyro-Glu (N-term Q) was set as dynamic modification. The tolerance of MS is 10 ppm and MS/MS is 0.5 Da. The criteria of minimal number of peptide per identified protein is 2.

### Animal

Experiments were performed on seven-week-old littermate ICR (CD1) mice with a defined weight range (30–35 g). Mice were maintained in specific pathogen-free conditions. They were housed in a 12:12 hour light dark cycle at a temperature of 22°C and a humidity level of 60–70%. Animals had ad libitum access to food and water.

### Animal ethics statement

Experiments involving the care and injection of mice with various venoms were reviewed and approved by the Institutional Animal Care and Use Committee of Chang Gung University (Permit Number: CGU106-194). The protocol of animal study on mice was based on the guidelines given by the law of animal protection act in Taiwan and the Council for International Organizations of Medical Sciences (CIOMS) [22].

### The median lethal dose (LD_50_) assay

Groups of 5 mice are subcutaneously injected, in the dorsal skin, with 0.1 ml of sterile saline solution containing different doses of venom (0.2–0.45 mg/kg). LD_50_ was determined by recording deaths 24 hours after injection, and the value of LD_50_ is estimated with Probit analysis [23]. One venom LD_50_ is defined as the minimal amount of venom causing death in 50% of the mice.

### The minimal necrosis dose (MND) assay

Groups of 3 mice were intradermally injected, in the dorsal region, with varying amount (0–60 μg) of *N*. *atra* venom (or cytotoxins), dissolved in 0.05 ml sterile saline solution. Animals were sacrificed by CO_2_ inhalation three-day post-injection. The dorsal skin was then removed and the necrotic lesion in the inner side of the skin was measured, and the MND is estimated with linear regression analysis. The MND was defined as the dose which induced an area of necrosis with 5 mm diameter three days after injection [24].

### The median effective dose (ED_50_) assay

This test involves incubation of a challenge dose, 5 LD_50_, of venom with different volumes (0.25–5 μl) of the antivenom, adjusted to a constant volume (0.1 ml) with saline solution. The mixtures were incubated for 30 minutes at 37°C, then 0.1-ml aliquots of each mixture were injected subcutaneously into groups of mice (n = 5/group). Mice in the control group were injected with a saline solution containing the challenge dose of venom alone, which induces 100% lethality. ED_50_ was determined by recording deaths 24 hours after injection, and the value of ED_50_ was calculated using probit analysis. One ED_50_ is defined as the ratio of amount of venom to the volume dose of antivenom that keep 50% alive of mice. Another term called “potency”, expressed as the amount of venom that is completely neutralized per milliliter of antivenom, was calculated as previously described [25, 26].

### The MND-median effective dose (MND_50_) assay

The test is carried out as above, using 3 mice per group. 1.5 MND of venom (or cytotoxins) is selected as the challenge dose. The challenge dose was mixed with different doses of FNAV in 0.05 ml saline solution and incubated at 37 °C for 30 minutes, and the control group including venom solutions incubated with physiological saline solution alone. The mixture was then intradermally injected in the dorsal skin of lightly anaesthetized mice. The diameter of necrotic lesions is quantified 3 days after injection. The neutralizing ability of antivenom, expressed as MND-median effective dose (MND_50_), is estimated as the volume of antivenom which reduces the diameter of necrotic lesions by 50% when compared with the diameter of the lesion in mice injected with the control venom/saline mixture. The antivenom was considered ineffective when none of mice, administered with the maximum amount of antivenom, survived.

### Histological analysis

Mice were intradermally injected, as described above, with saline solution and 1.5 MND of CTXs, respectively. Animals were sacrificed by CO_2_ inhalation three-day post-injection. The dorsal skin was then removed and immediately placed in 10% formaldehyde for 24 h. After routine processing and embedding in paraffin, 3 μm sections were obtained and stained with hematoxylin and eosin (HE) for histological observation. All samples were analyzed with a light microscope.

### Envenoming and rescue rodent models

Groups of 3 mice were intradermally injected, in the dorsal region, with a mixture of antivenom (40 μl) and varying amount (80–160 μg) of *N*. *atra* venom. The time-course of survival rate was recorded each half hour in the 12-hour period. The surviving mice were additionally fed for 60 hr and sacrificed by CO_2_ inhalation. The dorsal skin was then removed and the necrotic lesion in the inner side of the skin was measured.

### Statistical analysis

Statistical analysis was performed using one-way ANOVA followed by Tukey’s Multiple comparison test, and t-tests. All statistical analyses were performed using Graphpad Prism 5 software (La Jolla, California, USA). Differences were considered statistically significant when *p*-value ≤ 0.05.

## Results

### Description and preparation of major protein components from *N*. *atra* venom

**Fig 1:**
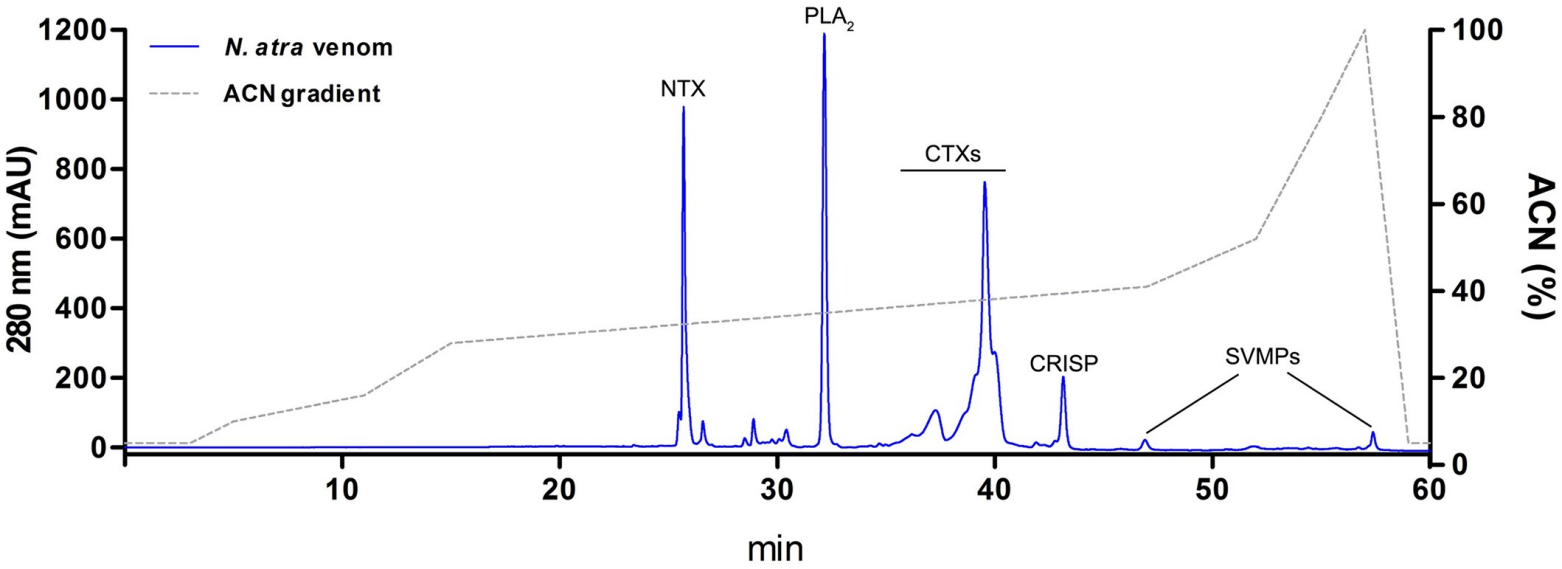
RP-HPLC fractionation of venom components from *N*. *atra*. Venom powder (100 μg protein) was dissolved in 0.1% formic acid and subjected to RP-HPLC analysis. Major proteins in resolved fractions were identified by LC-MS/MS. The identity of major protein components is indicated above each HPLC-separated fraction.

The crude venom of *N*. *atra* was separated by RP-HPLC (Fig 1). Consistent with previous studies using similar methods [15, 19], we purified and collected five major protein components. To confirm the identity of protein components in each fraction, we performed in-solution digestion together with LC-MS/MS. These results are summarized in S1 Table. The five major peaks in the RP-HPLC spectrum were identified as neurotoxin (NTX), phospholipase A_2_ (PLA_2_), cytotoxins (CTXs), cysteine-rich secretory protein (CRISP), and snake venom metalloproteinase (SVMP) (Fig 1).

**Fig 2:**
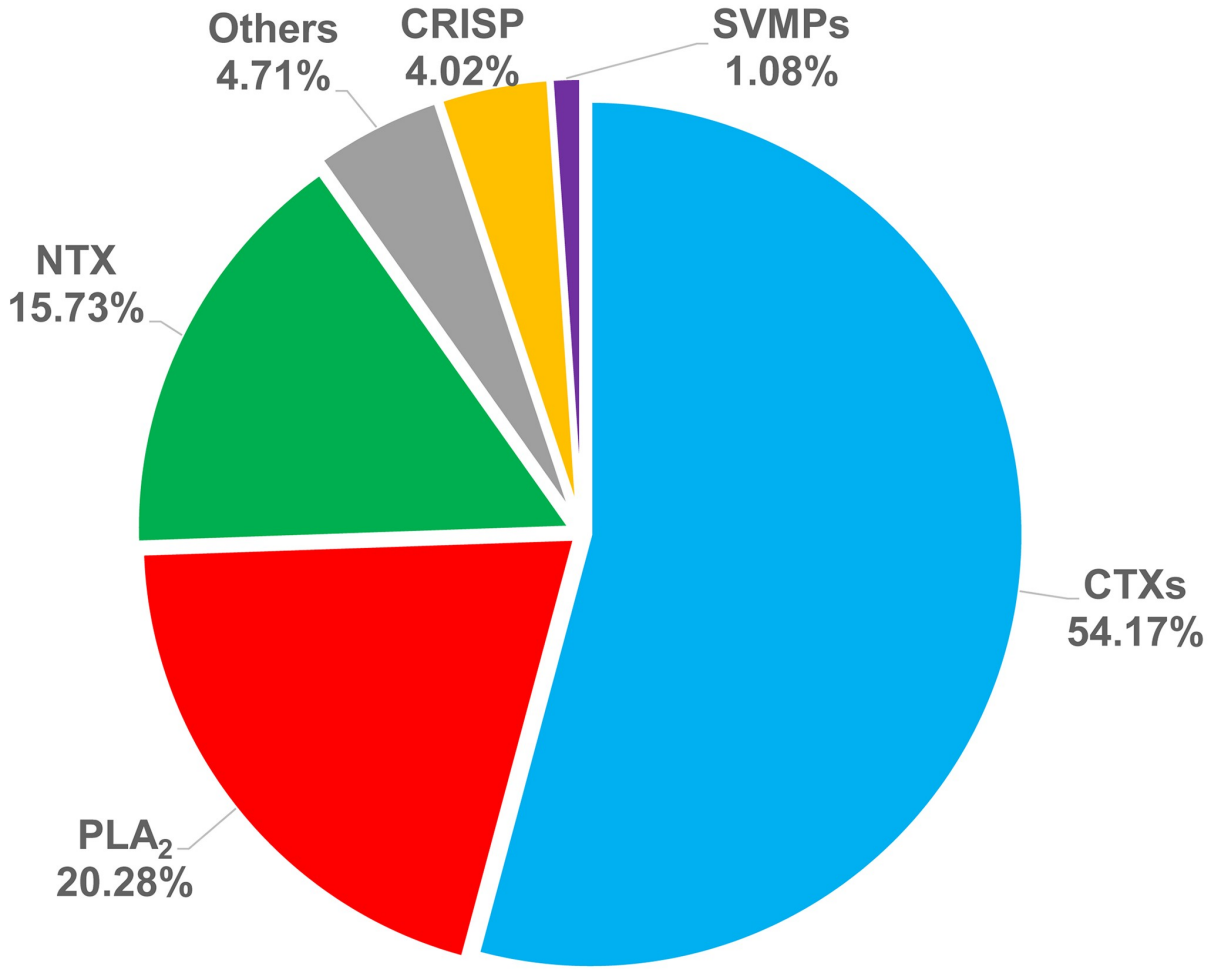
The relative abundance of venom components in *N*. *atra* venom. The relative abundance of each protein was calculated based on the peak area (280 nm) in RP-HPLC.

The relative abundance of these toxic components was calculated based on the absorbance intensity of their peaks in the RP-HPLC spectrum (Fig 2). More than half (54.2%) of *N*. *atra* venom consisted of CTXs. PLA_2_, NTX, CRISP, and SVMP constituted 20.3%, 15.7%, 4.0%, and 1.1%, respectively, of the total; the remaining 4.7% of venom proteins were other components. Collectively, CTX, PLA_2_, and NTX accounted more than 90% of protein components of *N*. *atra* venom. It has been claimed that CTXs are the major components that contribute to local necrosis [27]. To obtain a large amount of CTXs for further studies, we used HPLC methodology to purify 7 mg of CTXs from 15 mg of crude *N*. *atra* venom.

### Immuno-recognition of *N*. *atra* venom by FNAV

**Fig 3:**
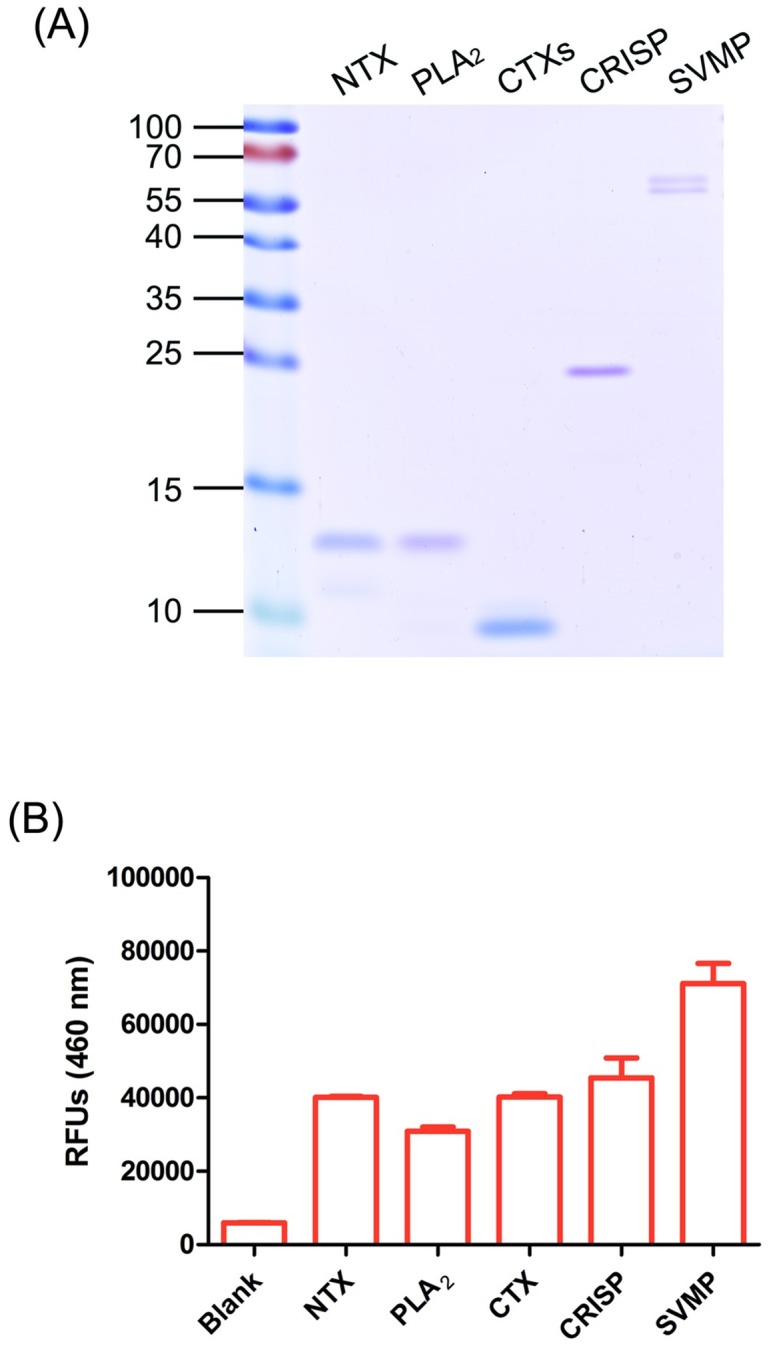
Immuno-profiling of FNAV against major components in *N*. *atra* venom. (A) Each component was analyzed by resolving 1 μg protein by SDS-PAGE. Gel bands were visualized by Coomassie Blue staining. (B) An equal amount of each protein was coated on 96-well plates and subjected to indirect ELISA using FNAV as the detecting antibody. The bar chart shows signals (means ± SD) from triplicate determinations.

To characterize fractionated venom components, we resolved proteins by sodium dodecyl sulfate-polyacrylamide gel electrophoresis (SDS-PAGE) and performed Coomassie Blue staining (Fig 3A). NTX, PLA_2_, CTXs and CRISP fractions each yielded a single major band on gels. The SVMP fraction appeared as two protein bands corresponding to molecular weights (MW) of 55 and 70 kDa. Results of MW and LC-MS/MS analyses (S1 Table) suggested that the upper and lower bands were zinc metalloproteinase disintegrin-like atragin (D3TTC2) and metalloproteinase disintegrin-like kaouthiagin-like (D3TTC1), respectively. The MWs of all protein bands coincided with the theoretical MWs based on protein identification results (S1 Table).

To evaluate and compare the titer of FNAV against each venom protein, we performed indirect enzyme-linked immunosorbent assays (ELISAs) using FNAV as the detection antibody (Fig 3B). FNAV showed the ability to recognize all five major venom components from *N*. *atra*. Surprisingly, the titer of FNAV toward SVMP was highest, even though the relative abundance of SVMP was the lowest of these five toxins. FNAV-interaction signals were almost equal for NTX, CTXs and CRISP, which did not significantly differ among each other, and the titer against PLA_2_ was the lowest.

### Development of an animal model of CTX-induced necrosis

**Fig 4:**
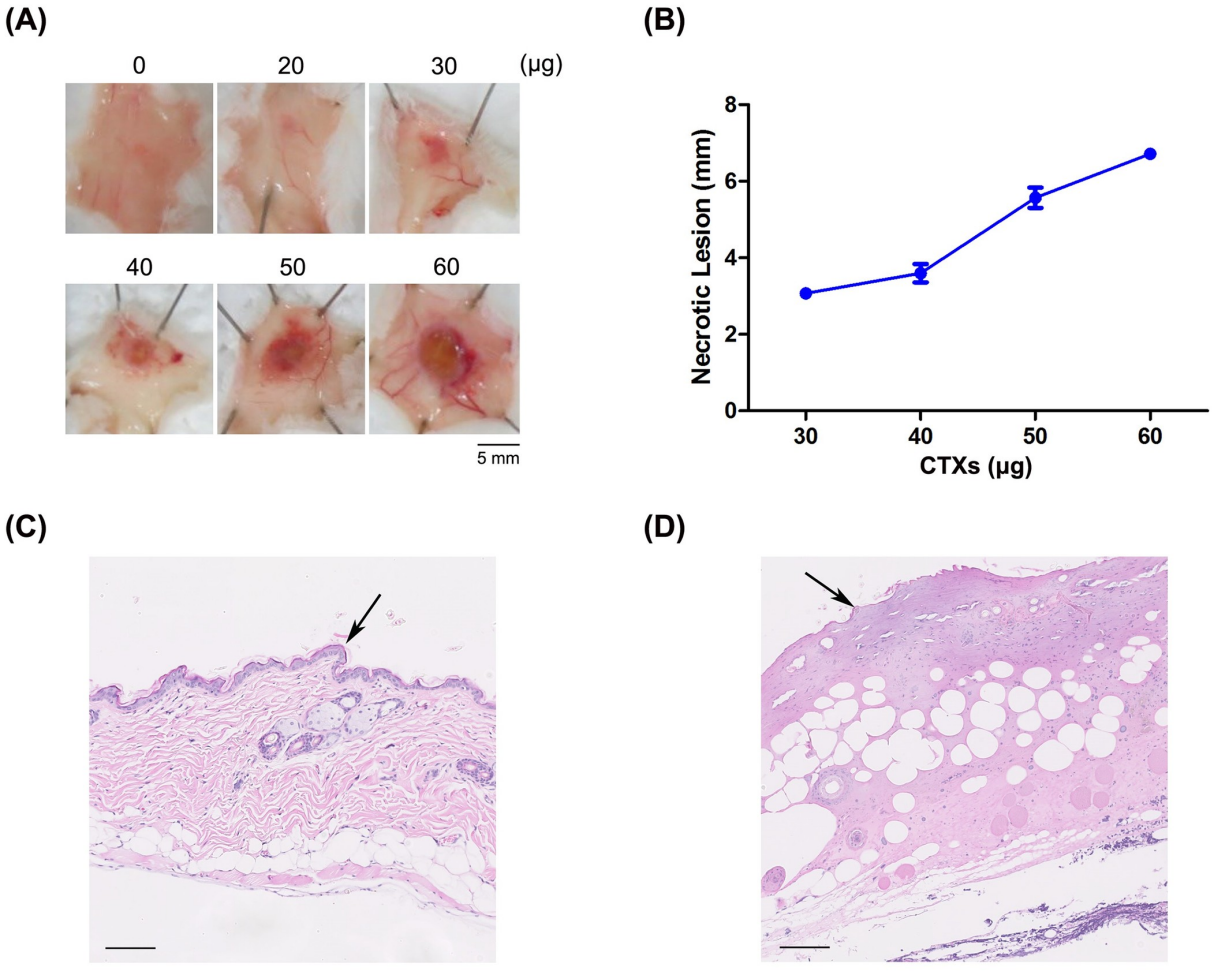
CTX-induced necrosis in mouse models. (A) Different amounts (0–60 μg) of CTXs were intradermally injected into mice, and the sizes of necrotic lesions in the dorsal skin of injected mice were measured. (B) MNDs for CTXs, calculated from measured sizes of necrotic lesions. H&E-stained sections of dorsal skins injected with (C) saline solution or (D) CTXs were analyzed histologically under a light microscope. Arrows highlight alterations in the epidermis. Scale bar: 100 μm.

To evaluate the necrosis-inducing activity of venom *in vivo*, we sought to develop a dermo-necrosis mouse model, followed WHO criteria for biological standardization [7]. First, we administered whole *N*. *atra* venom to mice to induce necrosis in the dorsal skin. Intradermal injection of mice with a total amount of venom greater than 30 μg resulted in the death of all mice within 12 hours. However, no necrotic lesions were observed in the dorsal skin following injection of whole venom. We next tested administration of purified CTXs. Necrotic lesions were observed in the dorsal skin following injection of more than 30 μg CTXs (Fig 4A), and the size of necrotic lesions increased with increasing CTX dose (Fig 4B). Calculations showed that the minimum necrotizing dose (MND) of CTX was 47 μg (Table 1). We also evaluated the 50% lethal dose (LD_50_) of whole cobra venom and purified CTX for subsequent assessment of neutralization ability. The LD_50_ of whole cobra venom was 0.29 μg/g; in contrast, mice survived injection of 100 μg (~3.3 μg/g) of CTXs (Table 1). Twitch, muscle weakness and respiratory paralysis occurred in venom-administrated mice, whereas these symptoms were not observed in CTX-administrated mice.

**Table 1:** Neutralization of toxic activities of *N*. *atra* venom by FNAV.

*N*. *atra*	Lethality	Necrosis	Neutralization ability of FNAV			
			Lethal ability		Necrotic ability	
	LD_50_ (μg/g)	MND (μg)	ED_50_ (mg/ml)	Potency (mg/ml)	MND_50_ (mg/ml)	Potency (mg/ml)
whole venom	0.29 (0.14–0.37)	NA	47.86 (30.17–110.56)	38.3	ND	
cytotoxins	NA ^a^	47 (43.9–50.3)	ND ^b^		NE ^c^	

Results were presented as means and 95% confidence limits.

^a^NA: no activity was detected.

^b^ND: neutralization ability not determined because proteins lacked activity.

^c^NE: neutralization activity was not affected by administration of the maximum dosage of FNAV.

To analyze histopathologic changes associated with CTX-induced necrosis, we evaluated sections of skin samples under a light microscope (Fig 4C and 4D & S1 Fig). Dorsal skin of mice injected with saline solution displayed normal epidermis and dermis histological structures (Fig 4C). In contrast, the epidermis was loosened and formed a hyaline fibrinoid material in skin samples from CTX-injected mice, and there was obvious evidence of inflammatory infiltrate in the dermis (Fig 4D).

### Evaluation of the ability of FNAV to neutralize CTX-induced necrosis

**Fig 5:**
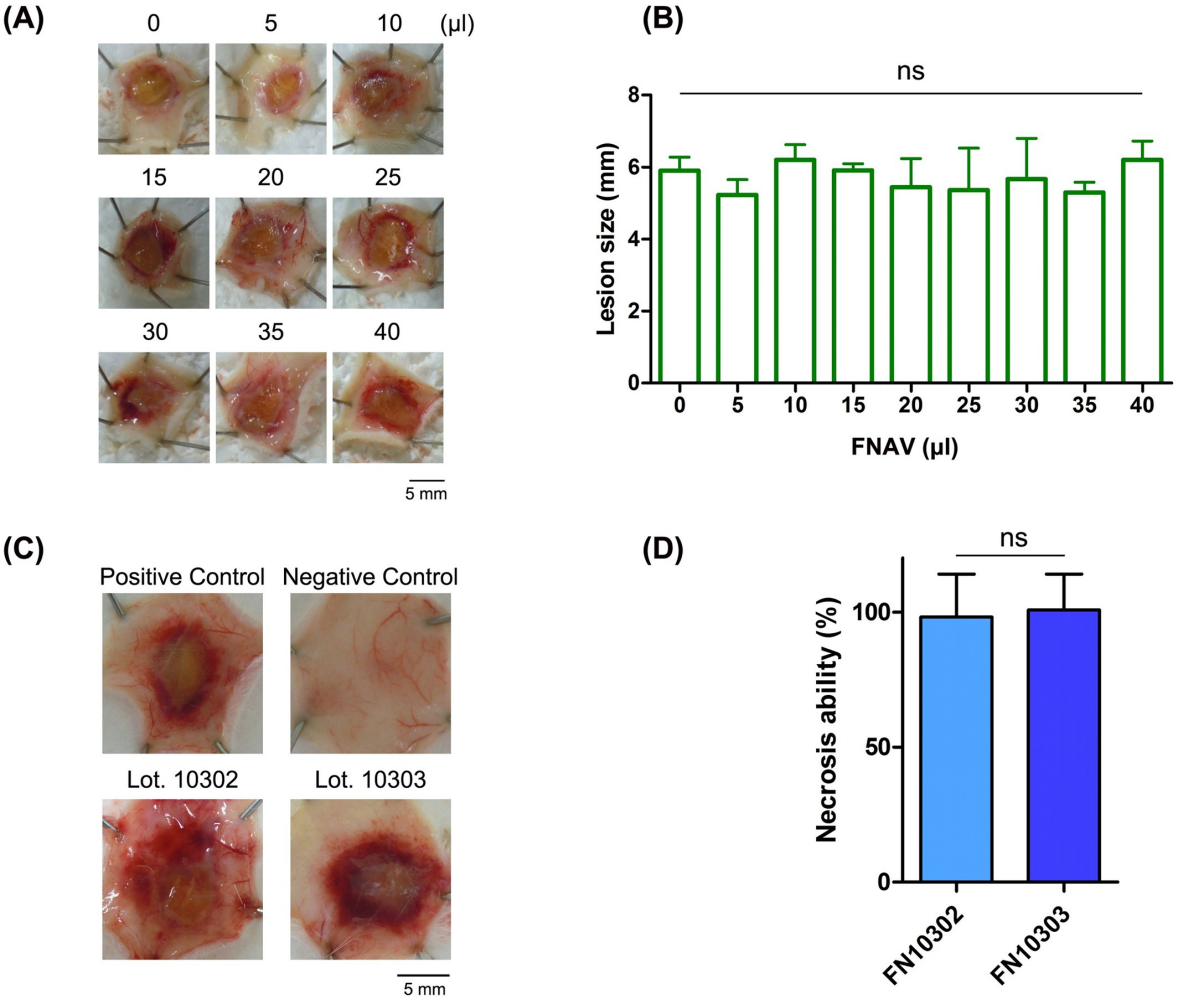
Evaluation of the ability of FNAV to neutralize CTX-induced necrosis. (A) Different volumes of FNAV (0–40 μl) were pre-incubated with a fixed amount of CTXs (1.5 MND), and then intradermally injected into mice. The sizes of necrotic lesions in the dorsal skin of injected mice were measured. (B) Average size of necrotic lesions (n = 3) after neutralization by different volumes of FNAV. One-way ANOVA followed by Tukey’s multiple comparison test was performed to analyze differences. (C) The sizes of necrotic lesions were recorded after mice had been administered two additional batches of FNAV (40 μl). Positive controls were injected with 1.5 MND of CTXs, and negative controls received the same volume of PBS. (D) Necrosis-inducing ability presented as the size of necrotic lesions compared with that of positive controls. Each bar represents means ± SD of triplicates (ns: not significant; two sample t-test).

Next, we used the CTX-induced necrotic mouse model to assess the potential of FNAV to prevent necrosis. In these experiments, antivenom was pre-incubated with CTXs before injection. Surprisingly, dermo-necrosis was still observed following administration of the highest volume of FNAV (40 μl) (Fig 5A). Moreover, the size of necrotic lesions did not decrease with increasing volumes of added FNAV and remained larger than 5 mm in diameter (Fig 5B). By contract, FNAV successfully neutralized the lethality of whole cobra venom, with an ED_50_ of ~47.9 mg/ml (Table 1).

These data seem to indicate that FNAV has limited potential for preventing the occurrence of local necrosis. However, antivenoms exhibit batch-to-batch variation, and individual horses in which FNAV was raised may have different immune responses toward CTXs, resulting in differences in antibody titer against CTXs. Thus, two additional batches of FNAV, FN1302 and FN1303, were selected and their ability to prevent the CTX-induced necrosis was accessed. Western blotting was performed to confirm that these batches of FNAV were active and able to recognize *N*. *atra* venom (S2 Fig). Again, FNAV (40 μl) from each batch was preincubated with 1.5 MND of CTX for 30 minutes, and then intradermally injected into the dorsal skin of mice. As shown in Fig 5C, necrotic lesions were observed in mice 3 days after injection. The sizes of necrotic lesions did not differ significantly compared with that in positive controls injected with 1.5 MND of CTX alone (Fig 5D).

### Development of an animal model of whole venom-induced necrosis

**Fig 6:**
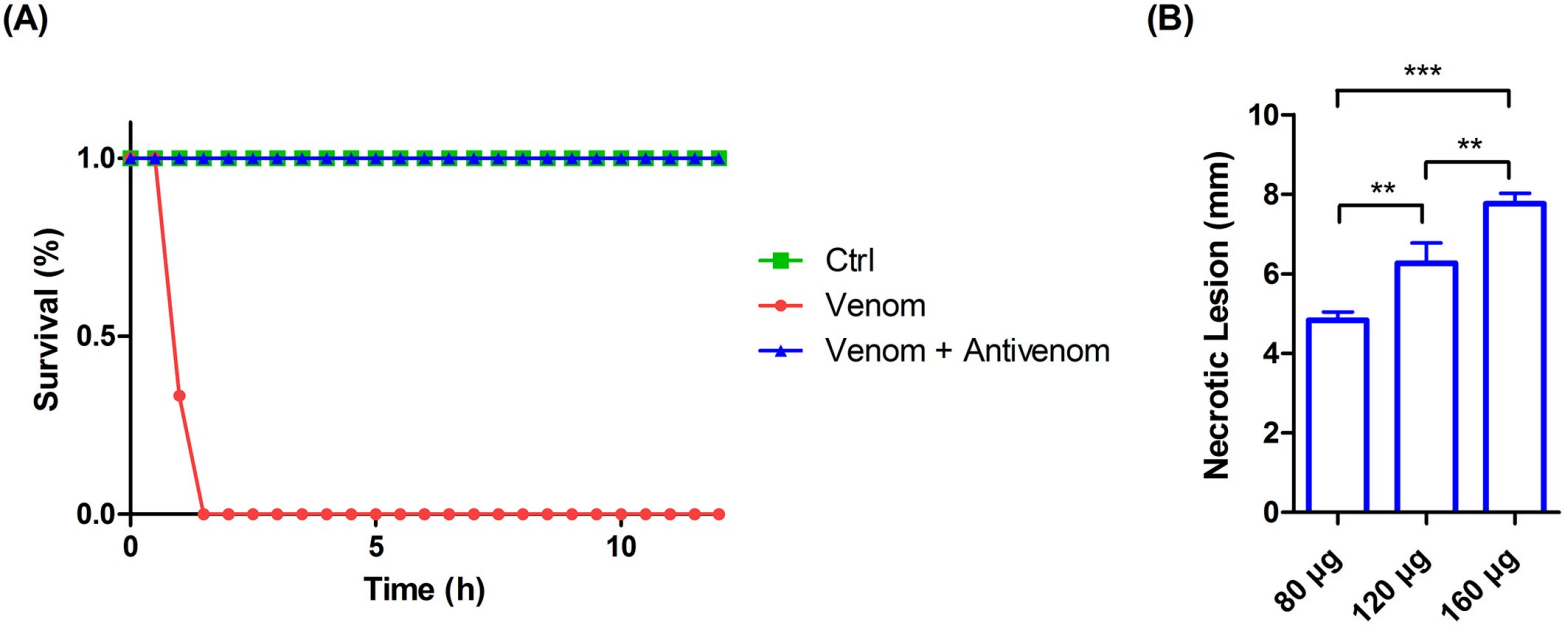
Development of local necrosis in mice administrated whole *N*. *atra* venom and FNAV. (A) The protective effect of FNAV was evaluated in mice. Mice (n = 3 per group) were administrated PBS (Ctrl), 80 μg of *N*. *atra* venom (Venom), or 80 μg of *N*. *atra* venom plus 40 μl of FNAV (Venom + Antivenom). The survival rate of mice was recorded every half-hour for 12 hours. (B) The sizes of necrotic lesions in mice that survived for 72 hours were measured and recorded. Each bar represents means ± SD of triplicates (*, *p* ≤ 0.05; **, *p* ≤ 0.01; ***, *p* ≤ 0.001; one-way ANOVA followed by Tukey’s multiple comparison test).

As described above, our original attempt to induce necrosis in the dorsal skin of mice by intradermally injecting the whole *N*. *atra* venom failed because all mice died within 12 hours after injection. Our finding that FNAV could neutralize the lethality of CTX, but could not prevent the CTX-induced necrosis in mice (Fig 5), raises the intriguing possibility of creating a whole venom-induced necrosis mouse model by simultaneously injecting whole *N*. *atra* venom and FNAV into mice at appropriate amounts and ratios. In developing snakebite animal models and assessing the ED_50_ of FNAV, researchers generally use 2.5–5 LD_50_ of *N*. *atra* venom as a challenge dose. However, our venom proteome (Fig 2) and lethality test results (Table 1) indicate that 5 LD_50_ of *N*. *atra* venom corresponds to about 20 μg of CTXs, which is incapable of inducing overt necrotic lesions. Thus, we used 80 μg, or approximately 9 LD_50_, of cobra venom as a challenge dose in the absence or presence of 40 μl FNAV. In the time-course experiments (Fig 6A), all mice in the control group (PBS injected) and the group administrated both cobra venom and FNAV survived the initial 12-hour period, whereas mice administered only cobra venom did not survive more than 1.5 hr. An examination of surviving mice in the cobra venom plus FNAV group, maintained on a normal diet for an additional 60 hours, revealed obvious dorsal skin necrosis. (S3 Fig). We also tested two groups of mice that were challenged with increased doses of cobra venom (120 and 160 μg) in the presence of FNAV. All mice in both groups survived and developed dorsal skin necrosis 3 days after injection (S3 Fig), and the average size of necrotic lesions increased in a challenge dose-dependent manner (Fig 6B).

## Discussion

Snakebite accidents involving *N*. *atra* are important in an emergency medicine setting in Taiwan. In this study, we characterized the venom proteome of *N*. *atra*, isolated necrosis-related CTX components, and evaluated the neutralization ability of FNAV—the clinically therapeutic antivenom for *N*. *atra* envenoming—against CTX-induced necrosis. Our results showed that more than half of *N*. *atra* venom proteins are CTXs. These components are the key cause of necrosis, which contributes to pathogenic complications in *N*. *atra* envenomation victims. Despite this contribution, our data showed that CTXs alone are not lethal (Table 1), suggesting that death observed in mice is predominantly caused by other toxic components. Furthermore, although CTXs were found to be immunogenic, such that FNAV was able to recognize CTXs in native form (Fig 3), antibodies in FNAV were unable to prevent the progression of CTX-induced necrosis. These results raise critical issues regarding the need for further discussion and improvement in antivenom production and evaluation of therapeutic efficacy.

CTXs, PLA_2_ and NTX account for ~90% of venom protein constituents [15, 19]. The structure of CTXs, the most dominant proteins in *N*. *atra* venom, is characterized by a three-fingered fold comprising loops I-III [28]. The tips of loop I-III form a hydrophobic core flanked by polar residues, which represent a membrane-binding motif [29–31]. This motif allows CTXs to bind cell membranes, an interaction that has been reported to cause a conformational change in the membrane and structural deformation of lipid bilayers [32, 33]. Several *in vitro* studies using model membranes or cell lines have suggested that CTXs are the major toxins that contribute to necrosis—a major pathological consequence of cobra envenomation [29, 30, 34–36]. In the current study, we used mouse models to further confirm that, although the pathological activity of CTXs is related to the symptom of local necrosis, CTX toxicity is not a determining factor in the fatality of cobra envenomation.

Although *N*. *atra* belongs to the *Elapidae* family, neurotoxic symptoms are not a clinically significant consequence of *N*. *atra* envenomation, unlike the case for other members of this family [11]. This is somewhat surprising, given that our investigation found that NTX constitutes about 15% of venom components of Taiwanese cobra. Importantly, LD_50_ assays reported here found that mice developed muscle weakness and respiratory paralysis after subcutaneous injection of *N*. *atra* venom, neurological effects that were also previously documented in mice [37, 38]. The reason for the discrepancy between neurotoxic effects of *N*. *atra* envenomation in mice and those in humans is still unclear. The NTX in *N*. *atra* venom is a short-chain α-neurotoxin. α-Neurotoxins are competitive antagonists that bind to nicotinic acetylcholine receptors (nAChR) in post-synaptic membranes, causing a neuromuscular-blocking effect [39–41]. The α-subunit of nAChR plays a critical role in α-neurotoxin binding, and the protein sequence of loop C in the α-subunit shows few amino acid differences between human and mouse [42, 43]. Furthermore, the inhibition of human nAChR induced by α-elapitoxin, a short chain α-neurotoxin from *Pseudechis porphyriacus*, was shown to be completely reversible within 6 minutes, a rate that is significantly faster than that for mouse nAChR [43]. This suggests that species-specific differences in nAChR underlie differences in neurotoxic effects between rodents and humans, and may be the reason for discrepancies in the pathogenesis of *N*. *atra* envenoming.

PLA_2_ constitutes about 20% of *N*. *atra* venom proteins (Fig 2), indicating that it may be an important factor in the pathogenesis of cobra envenoming. PLA_2_ from *N*. *atra* has been shown to exhibit strong binding activity toward zwitterionic membranes [44]. Additionally, PLA_2_ from *Naja kaouthia* venom was reported to promote CTX-induced cytotoxicity in cell models [45], indicating that cobra PLA_2_ may play a role in the pathogenesis of necrosis. Although the relative abundances of SVMP and CRISP are lower than those of the three major components in *N*. *atra* venom, they may contribute to the pathogenesis of cobra envenoming. SVMPs have the ability to inhibit classical and alternative pathways of the complement system by cleaving complement factors [46], and activating mast cells [47], indicating that the effect of SVMPs in cobra venom may act to disturb the immune system in local tissues. CRISP targets various ion channels, and weakly blocks muscle contraction evoked by potassium ions, similar to the weak toxicity exhibited by other snake venom cysteine-rich proteins [48, 49]. Despite considerable previous research effort, the detailed roles of *N*. *atra* PLA_2_, SVMP, and CRISP in clinical sequelae among snakebite victims remains unclear and should be further elucidated.

Antivenom for snakebite treatment, produced using horses, has been available in Taiwan since the 1920s [37]. Our ELISA results showed that the horse bivalent antivenom, FNAV, has the ability to recognize all five major components of *N*. *atra* venom (Fig 3B). Cobra venom exhibits high immunogenicity in a horse model, despite co-immunization with *B*. *multicinctus* venom. Here, we found that FNAV was able to neutralize cobra venom lethality in a mouse model with high potency, but lacked the ability to prevent CTX-induced necrosis. It is unclear why CTX-recognizing antivenom is unable neutralize CTX toxicity. One possible explanation is that FNAV-recognized epitopes are not the toxicity-related domains on CTXs, resulting in a lack of neutralization ability toward CTXs. Another possibility is that CTX is a low molecule weight protein (<10 kDa) exhibiting lower immunogenicity than other high molecule weight venom components [15, 50]. Immunization of crude cobra venom to prepare FNAV may cause the CTX-neutralizing antibodies are less than expected.

Currently, the suggested dosage of FNAV for treating envenomation by *N*. *atra* or *B*. *multicinctus* is based on the ability to reduce lethality in a mouse model. Our investigation suggests that the symptoms in venom-injected mice—primarily muscle weakness and respiratory paralysis—are attributable to NTX, whereas the major venom component, CTX, does not cause death when subcutaneously injected into mice. This indicates that NTX may be the major toxin in *N*. *atra* venom that contributes to rodent death. However, unlike observations in mice, cobra envenomation does not lead to life-threatening neurotoxicity in the clinic; instead, local necrosis—a hallmark of cobra snakebite—is usually the main symptom. Whether FNAV can neutralize CTXs and prevent the development of local necrosis was previously unclear. The results of the present study show that FNAV is not able to prevent the extension of local necrosis induced by CTX from *N*. *atra* venom (Fig 5). This raises critical issues about the efficacy tests used during antivenom production prior to submitting the antivenom for clinical use. Indeed, the rodent lethality-prevention assay is the WHO-recommended standard for testing the efficacy of snake antivenom before a production run is released [7]. However, snake venoms induce a variety of systemic pathological effects, including hemorrhage, pro-coagulopathy, anti-coagulopathy, neurotoxicity, myotoxicity and necrosis, among others. Moreover, not all pathologies are related to lethality. Sometimes, the antivenom has the ability to neutralize the lethal effects of a snake venom, but is unable to prevent or eliminate its most clinically relevant pathophysiological effects. Our study provides a good case in point, showing that FNAV did not prevent necrosis, a specific pathogenic feature induced by *N*. *atra* venom, despite being able to neutralize venom-induced lethality in a mouse model. Thus, supplementary assays to determine antivenom neutralization of specific venom-induced pathologies may be necessary for quality control of antivenom production.

Most cases of cobra envenoming will develop local necrosis [24, 51, 52]. Although this pathological effect is not life threating, it usually leads to an irreversible outcome for snakebite victims. Once the local necrosis occurs, patients will require debridement, fasciotomy, or even amputation. These surgeries permanently impact a patient’s life, and may further detrimentally affect a family if the victim is the primary source of income. Thus, although necrosis may not cause lethality, its consequences are far from trivial. Therefore, whether an antivenom is effective in protecting against the occurrence of local necrosis is a critical issue for countries where cobra snakebite is a constant threat. In the manufacture of antivenom for cobra envenoming, not only for *N*. *atra*, but also for other *Naja* species, we strongly recommend that manufacturers perform MND_50_ assays to determine whether a particular batch of antivenom is able to eliminate local necrosis or prevent expansion of the local necrosis—the most clinically relevant pathological effect induced by cobra venoms—before antivenoms are submitted to clinical use.

Given our finding that FNAV is, at best, only partially effective in controlling the local necrotic damage induced by *N*. *atra* venom, it is important to develop novel therapeutic interventions for treating *N*. *atra* envenomation in Taiwan. Several advanced biotechnological tools have recently been adopted for developing alternative strategies for antivenom production [53, 54]. Applying expertise in synthetic biology, antibody research and immunology, a number of researchers have attempted to develop recombinant antivenoms and investigate their ability to neutralize toxins from snake venom [55–59]. Recombinant antivenoms containing several monoclonal antibodies or single-chain variable fragments (scFv) that target CTXs may be a promising source material for solving the problem of *N*. *atra*-induced local tissue damage. A previous study reported a situation similar to our case [56], showing that the currently used equine polyvalent antivenom exhibited a limited ability to neutralize dendrotoxin-mediated neurotoxicity. In this case, the researchers developed a cocktail of a few humanized monoclonal antibodies against this neurotoxin. We could follow these authors’ strategy to screen for anti-CTX monoclonal antibodies that are capable of preventing venom-induced local necrosis extension. These anti-CTXs monoclonal antibodies could them be combined with current FNAV to serve as a novel recombinant antivenom for treatment of cobra envenomation in Taiwan. This recombinant antivenom would not only conserve the original potential of FNAV to neutralize cobra-induced lethality, it would also have the additional ability to prevent the occurrence of local tissue necrosis. In addition, a novel hydrogel nanoparticle that inhibits *Naja nigricollis* venom-induced dermo-necrosis *in vivo* has been reported [60]. These engineered nanoparticles bind to PLA_2_ and three-finger toxins from *Elapidae* snakes and sequester them, preventing them from interacting with their membrane targets and thereby inhibiting their biological activities. This specific therapeutic application demonstrates the potential for inhibiting the cytotoxicity and dermo-necrosis ability of African spitting cobra venom in cells and animal models. It may be an alternative to solve the problem of local necrosis induced by cobra envenomation in Taiwan.

Whether FNAV has the potential to prevent local necrosis extension induced by *N*. *atra* venom has been debated for more than a decade in Taiwan [9, 11–14]. Following the standard protocol described previously [61, 62] has failed to yield a mouse model of *N*. *atra* venom-induced necrosis for investigating the neutralization potential of FNAV *in vivo*. Venom-injected mice would die due to neurotoxicity before necrotic lesions developed. In the present study, we used RP-HPLC to purify CTXs—the necrosis-related toxins—to successfully develop a local necrosis mouse model. This resulting model is a good tool for evaluating the necrosis-neutralizing ability of antivenom, or other potential drugs, toward *N*. *atra* venom *in vivo*.

Despite having developed a necrotic animal model and used it to assess the ability of FNAV to neutralize cobra venom-induced necrosis, there are three limitations to our work. First, our strategy ignored direct or synergistic contributions of other cobra venom proteins to the pathogenesis of necrosis. As noted above, PLA_2_ proteins in *N*. *atra* venom may have synergistic effects that promote local necrotic damage [44, 45, 63]. Furthermore, clinical effects of other proteins in *N*. *atra* venom, such as CRISP and SVMP, are unclear, and may be involved in the pathogenesis of necrosis. Whether these proteins contribute to necrosis or other specific physiological processes associated with clinical symptoms should be further evaluated. Second, our results demonstrated that FNAV has limited potential for preventing the occurrence of local necrosis. Although three batches of FNAV were evaluated in our study, we should further evaluate additional batches or antiserum from different horses, to verify our findings. Finally, although pre-incubation of venom and antivenom before delivery to animal models is the WHO-recommended procedure for evaluating neutralization efficacy, these necrosis neutralization assays do not completely recapitulate the real circumstance of cobra snakebite. Accordingly, these experiments neglected to take into account the influence of the toxicokinetics of venoms and the pharmacokinetics of antivenom. Efficacy tests should be further investigated under conditions in which antivenom is administrated in mice after injection of venom so as to more accurately mimic the actual circumstances of snakebite.

Taken together, the data provided here show that isolated CTXs, the major necrosis-related proteins in *N*. *atra* venom, were able to produce dermo-necrosis in rodent models, but did not induce lethality, even at high doses. FNAV, the conventional antivenom for *N*. *atra* treatment in Taiwan, was highly potent in neutralizing the lethality of whole cobra venom; however, it was unable to prevent CTX-induced necrosis *in vivo*. Moreover, dermo-necrosis was still observed in mice following subcutaneous injection of a high dose of cobra venom mixed with FNAV, which imitated the clinical circumstance of cobra envenoming in Taiwan. This finding provides insights that should help improve current antivenoms and advance cobra envenoming management in Taiwan. In addition, our case provides an example in which supplementary quality control assays may be necessary to determine the effectiveness of antivenoms in neutralizing specific pathologies induced by the venom; evaluating only the ability to prevent rodent lethality is insufficient. Thus, characterizing and including supplementary quality control assays is encouraged in antivenom production worldwide, focusing on specific pathologies of snake envenoming.

## Supporting information

S1 Table Identification of major protein fractions in *N*. *atra* venom. (XLSX)

S1 Fig Microscopic images of hemorrhagic spots in the dorsal skin. H&E-stained sections of dorsal skin of mice injected with (A) saline solution or (B) CTXs were observed under a light microscope. Images correspond to an area near hemorrhagic spots. Scale bar: 100 μm.
(TIF)

S2 Fig Immunorecognition of *N*. *atra* venom by two different batches of FNAV. SDS-PAGE was performed to separate *N*. *atra* venom. After transferring *N*. *atra* venom proteins onto PVDF membranes, each lane was probed with FNAV batches FN1302 and FN1303.
(TIF)

S3 Fig Necrotic lesions in mice induced by whole *N*. *atra* venom in the presence of FNAV. Different amounts of *N*. *atra* venom were mixed with a fixed volume (40 μl) of FNAV. Each mixture was administrated into mice, and necrotic lesions in mouse dorsal skin were measured and recorded 72 hours after injection.
(TIF)
