# Supplementary figures and images for: Pathogenesis of local necrosis induced by Naja atra venom: Assessment of the neutralization ability of Taiwanese freeze-dried neurotoxic antivenom in animal models
Source: PLoS Negl Trop Dis. 2020 Feb 7;14(2):e0008054. doi: 10.1371/journal.pntd.0008054 (PMC7032728; doi:10.1371/journal.pntd.0008054)

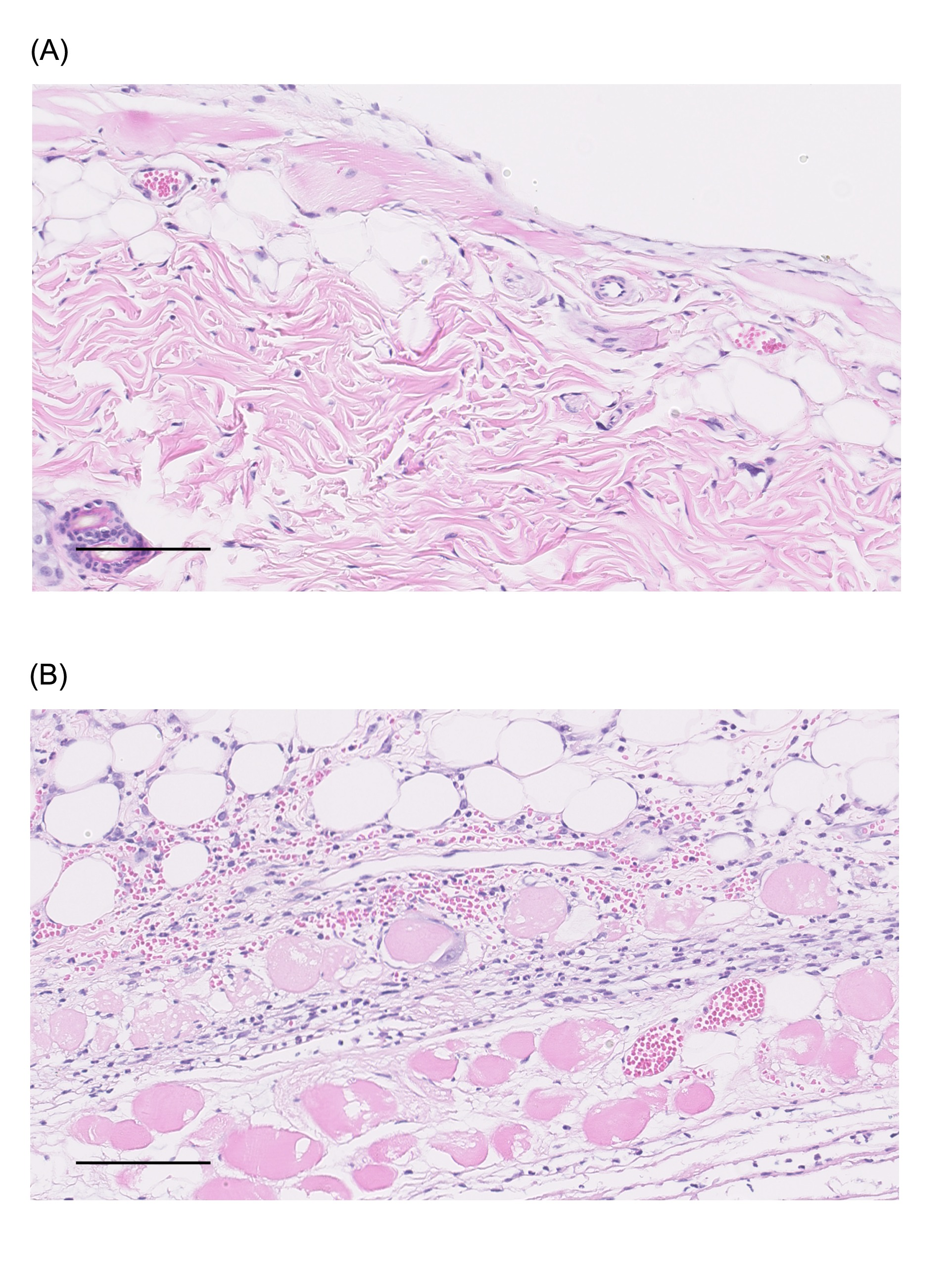

Supplement: S1 Fig — H&E-stained sections of dorsal skin of mice injected with (A) saline solution or (B) CTXs were observed under a light microscope. Images correspond to an area near hemorrhagic spots. Scale bar: 100 μm. (TIF) [file pntd.0008054.s002.tif]

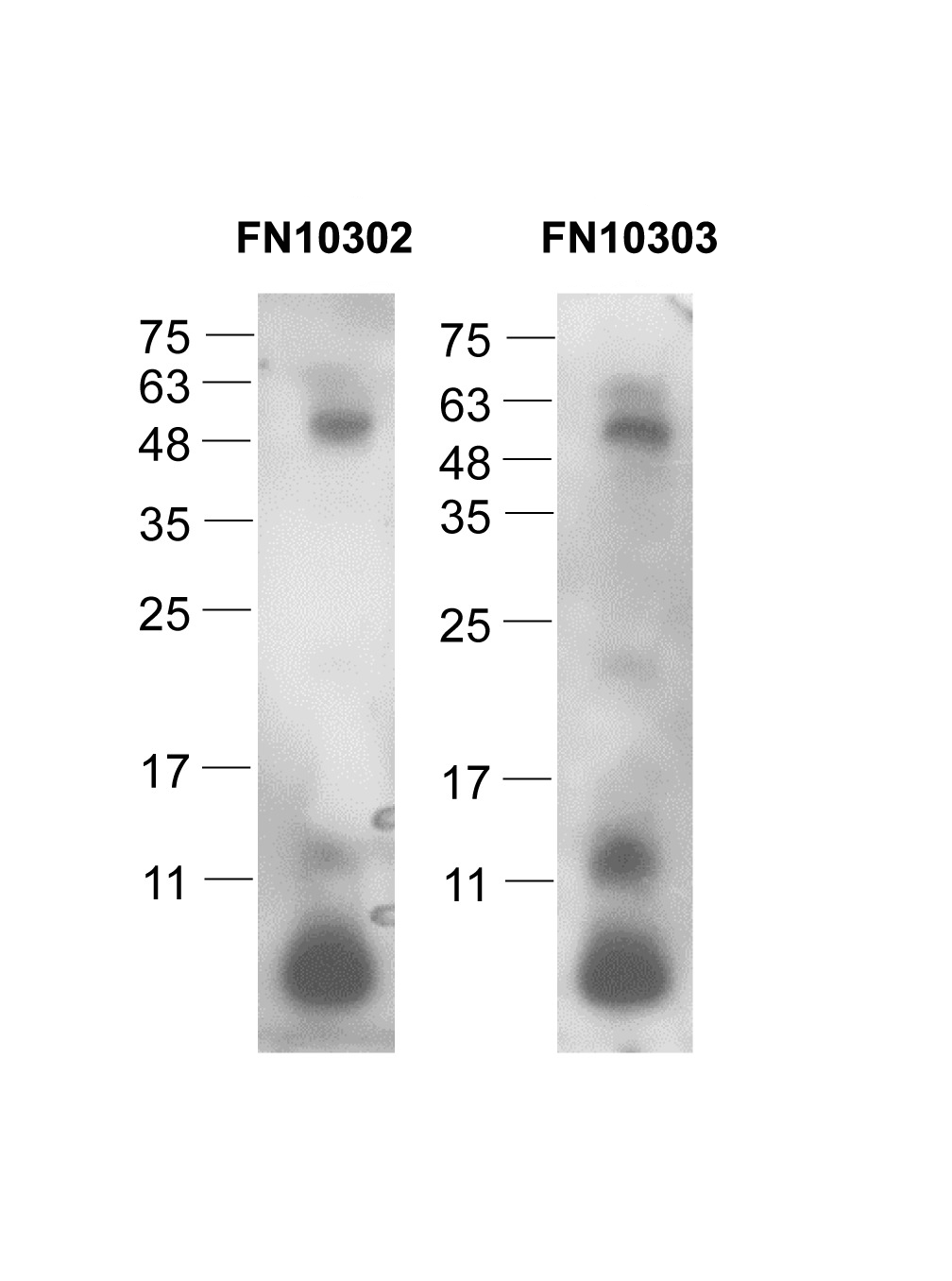

Supplement: S2 Fig — SDS-PAGE was performed to separate N. atra venom. After transferring N. atra venom proteins onto PVDF membranes, each lane was probed with FNAV batches FN1302 and FN1303. (TIF) [file pntd.0008054.s003.tif]

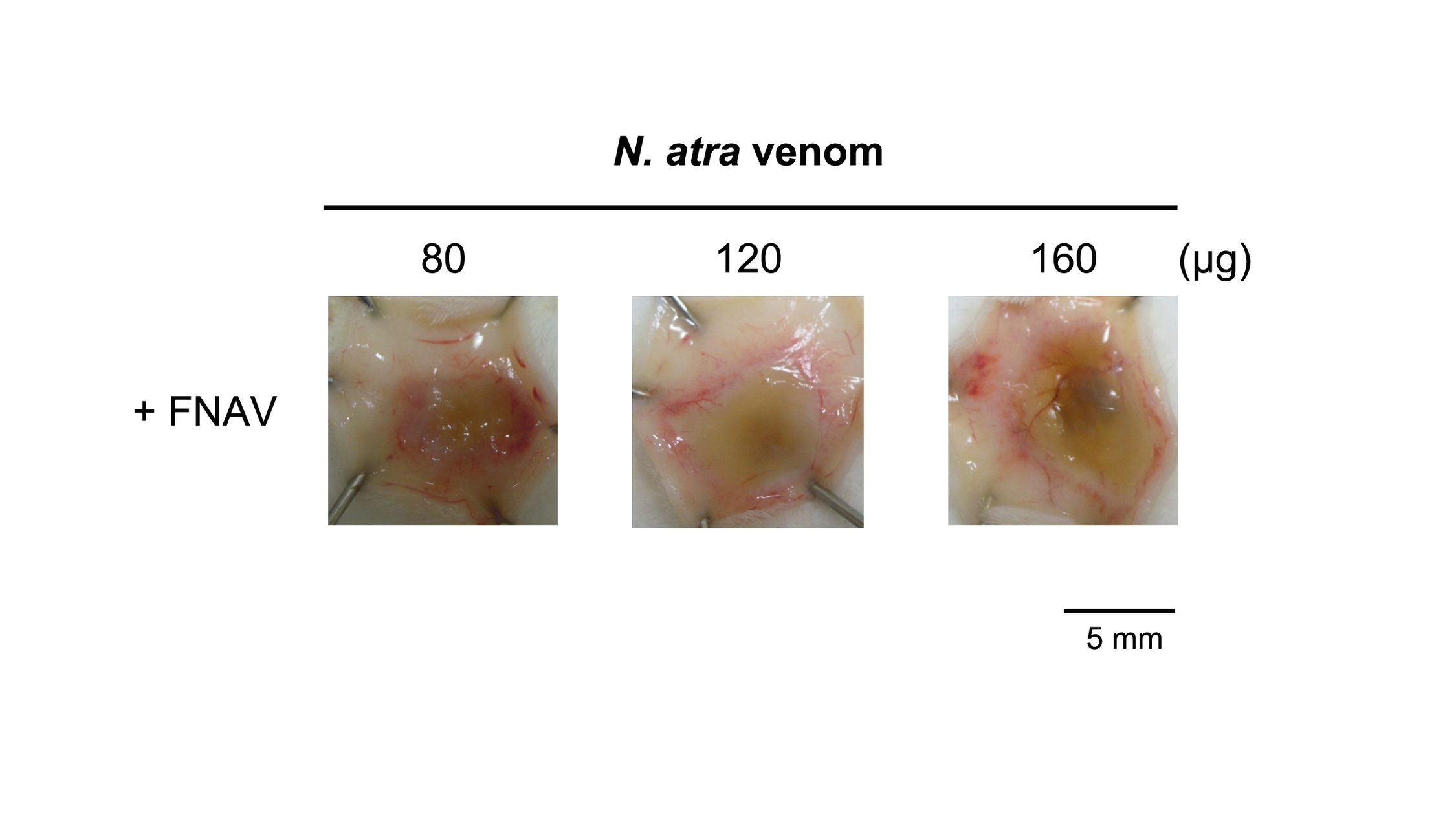

Supplement: S3 Fig — Different amounts of N. atra venom were mixed with a fixed volume (40 μl) of FNAV. Each mixture was administrated into mice, and necrotic lesions in mouse dorsal skin were measured and recorded 72 hours after injection. (TIF) [file pntd.0008054.s004.tif]
